# Obituary

**Published:** 1894-01

**Authors:** 


### Obituary.

ARTHUR WELLESLEY EDIS, M. D., London, F.R.C.P., died recently in London. From the *British Medical Journal*, of November 16th, we condense the following: Dr. Edis was born in Huntingdonshire in 1840, pursued his preliminary education in the grammar schools of Huntingdon and Aldenham, and a course of instruction in agriculture at the Cirencester College, winning honors in veterinary surgery. Thence he passed on to Westminister Hospital. He took the membership of the Royal College of Surgeons, of England, in 1862, and became a member of the Royal College of Physicians in 1867. For five years he was assistant physician to the Hospital for Women, Soho Square, subsequently taking the post of assistant obstetric physician to the Middlesex Hospital, and afterwards becoming physician and lecturer on diseases of women. At the time of his death he was senior physician to the Chelsea Hospital for women, and held also other public offices. He was past president of the British Gynecological Society, and honorary fellow of many foreign obstetrical and gynecological societies, among which was that of the American Association of Obstetricians and Gynecologists. His death is lamented throughout England, as well as in foreign countries, where he was well known.

